# Recombinant antigens used as diagnostic tools for lymphatic filariasis

**DOI:** 10.1186/s13071-021-04980-3

**Published:** 2021-09-15

**Authors:** André Filipe Pastor, Maressa Rhuama Silva, Wagner José Tenório dos Santos, Tamisa Rego, Eduardo Brandão, Osvaldo Pompilio de-Melo-Neto, Abraham Rocha

**Affiliations:** 1 Fundação Oswaldo Cruz-Fiocruz, Instituto Aggeu Magalhães, Recife, PE Brazil; 2 Instituto Federal de Educação, Ciência e Tecnologia do Sertão Pernambucano (IFSertao-PE), Campus Floresta, Floresta, PE Brazil; 3 Fundação Oswaldo Cruz-Fiocruz, Instituto Aggeu Magalhães, Serviço de Referência Nacional em Filarioses, Recife, PE Brazil; 4 Laboratório do Hospital Otávio de Freitas, Secretaria de Saúde do Estado de Pernambuco, Recife, PE Brazil

**Keywords:** *Wuchereria bancrofti*, ELISA, Antibodies, Sensitivity, Specificity

## Abstract

Lymphatic filariasis (LF) is a parasitic disease caused by the worms *Wuchereria bancrofti*, *Brugia malayi*, or *Brugia timori*. It is a tropical and subtropical illness that affects approximately 67 million people worldwide and that still requires better diagnostic tools to prevent its spread and enhance the effectiveness of control procedures. Traditional parasitological tests and diagnostic methods based on whole protein extracts from different worms are known for problems related to sample time collection, sensitivity, and specificity. More recently, new diagnostic tools based on immunological methods using recombinant antigens have been developed. The current review describes the several recombinant antigens used as tools for lymphatic filariasis diagnosis in antigen and antibody capture assays, highlighting their advantages and limitations as well as the main commercial tests developed based on them. The literature chronology is from 1991 to 2021. First, it describes the historical background related to the identification of relevant antigens and the generation of the recombinant polypeptides used for the LF diagnosis, also detailing features specific to each antigen. The subsequent section then discusses the use of those proteins to develop antigen and antibody capture tests to detect LF. So far, studies focusing on antibody capture assays are based on 13 different antigens with at least six commercially available tests, with five proteins further used for the development of antigen capture tests. Five antigens explored in this paper belong to the SXP/RAL-2 family (BmSXP, Bm14, WbSXP-1, Wb14, WbL), and the others are BmShp-1, Bm33, BmR1, BmVAH, WbVAH, BmALT-1, BmALT-2, and Wb123. It is expected that advances in research with these antigens will allow further development of tests combining both sensitivity and specificity with low costs, assisting the Global Program to Eliminate Lymphatic Filariasis (GPELF).

**Supplementary Information:**

The online version contains supplementary material available at 10.1186/s13071-021-04980-3.

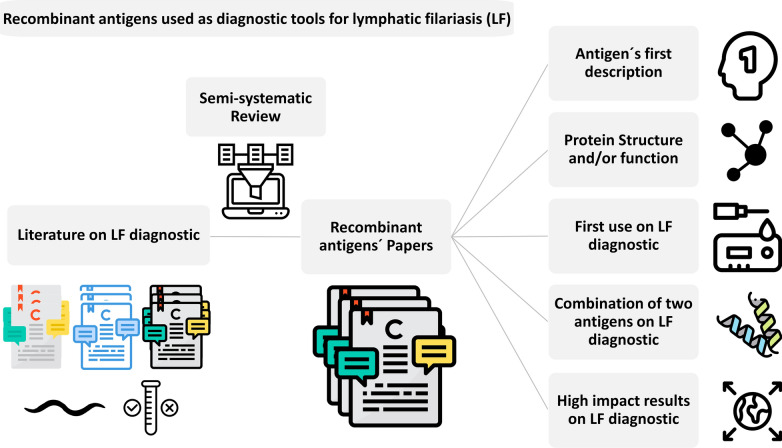

## Background

Lymphatic filariasis (LF) is an endemic tropical and subtropical parasitosis that affects approximately 67 million people worldwide. Also known as elephantiasis, in its chronic and symptomatic phase, it is caused by the nematode worms *Wuchereria bancrofti*, *Brugia malayi*, or *Brugia timori* [1]. LF is considered a major threat to public health, and its severe socioeconomic impact has been the subject of many studies in different endemic regions [2, 3]. Studies in India, for example, have estimated the average annual costs of treating adenolymphangitis and chronic cases as more than US$ 30 million [4]. The strong stigma attached to the afflicted individuals, combined with the physical disability, contributes to them being excluded from job opportunities [5]. In 1997, the World Health Organization (WHO) created the Global Programme to Eliminate Lymphatic Filariasis (GPELF), which aimed to eliminate LF by 2020. It has three main pillars: (i) interruption of transmission; (ii) assistance to people with morbid disease forms; and (iii) development of new and efficient diagnostic strategies [6]. The last should be used not only to identify specific cases of infection but also for the epidemiological surveillance of those individuals from areas undergoing mass treatment [7].

Parasitological diagnostic methods for LF are based on the visual detection of microfilaria from capillary and venous blood samples, using thick smear and membrane filtration techniques, respectively [8, 9]. In particular, the thick smear approach has been used worldwide for several decades because it is a low-cost technique that demands little infrastructure [10]. However, these tools alone should not define the infection status, especially in individuals who have low parasitemia or are amicrofilaremic despite being infected with adult worms [11]. Furthermore, to increase the sensitivity of these tests, blood samples must be collected at a time day that is compatible with the brugian and bancroftian microfilariae periodicity, which is adapted to the vector feeding behavior. For microfilaria with nocturnal periodicity, for example, the blood collection should be carried out between 10:00 p.m. and 02:00 a.m. [12].

Antibodies against filarial proteins are known to be sensitive markers of transmission intensity and can provide evidence of continued exposure to filarial infection, even before or after antigenemia or microfilaria detection. Individuals living in endemic regions have been reported to have a high proportion of immunoglobulin G4 (IgG4) antibodies against known filarial antigens, even if they do not have circulating microfilaria or detectable filarial antigens [13]. Seeking to meet the GPELF demands, new diagnostic tools based on immunological methods using recombinant antigens have been developed [14–16]. These were based on recombinant antigens either aiming to capture antibodies from sera of infected individuals or used to produce antibodies against specific filarial antigens which then can be used to directly capture the same antigens from the sera [17, 18]. The new tools have the advantage of higher sensitivity over parasitological methods and can be applied to samples collected at any time of the day. Also, they provide quick results and require minimal infrastructure [19, 20]. These assays are critical for the successful verification of LF elimination programs in areas under intervention, as they can provide the basis for an alert system assessing any further contact with infectious forms of the parasite. In the present article, we review the literature (Additional file 1: Text S1) on the main recombinant antigens used for LF diagnosis based on antibody and antigen assays, highlighting their advantages and limitations, as well as the commercial tests developed based on them.

## Recombinant antigens

**Fig. 1:**
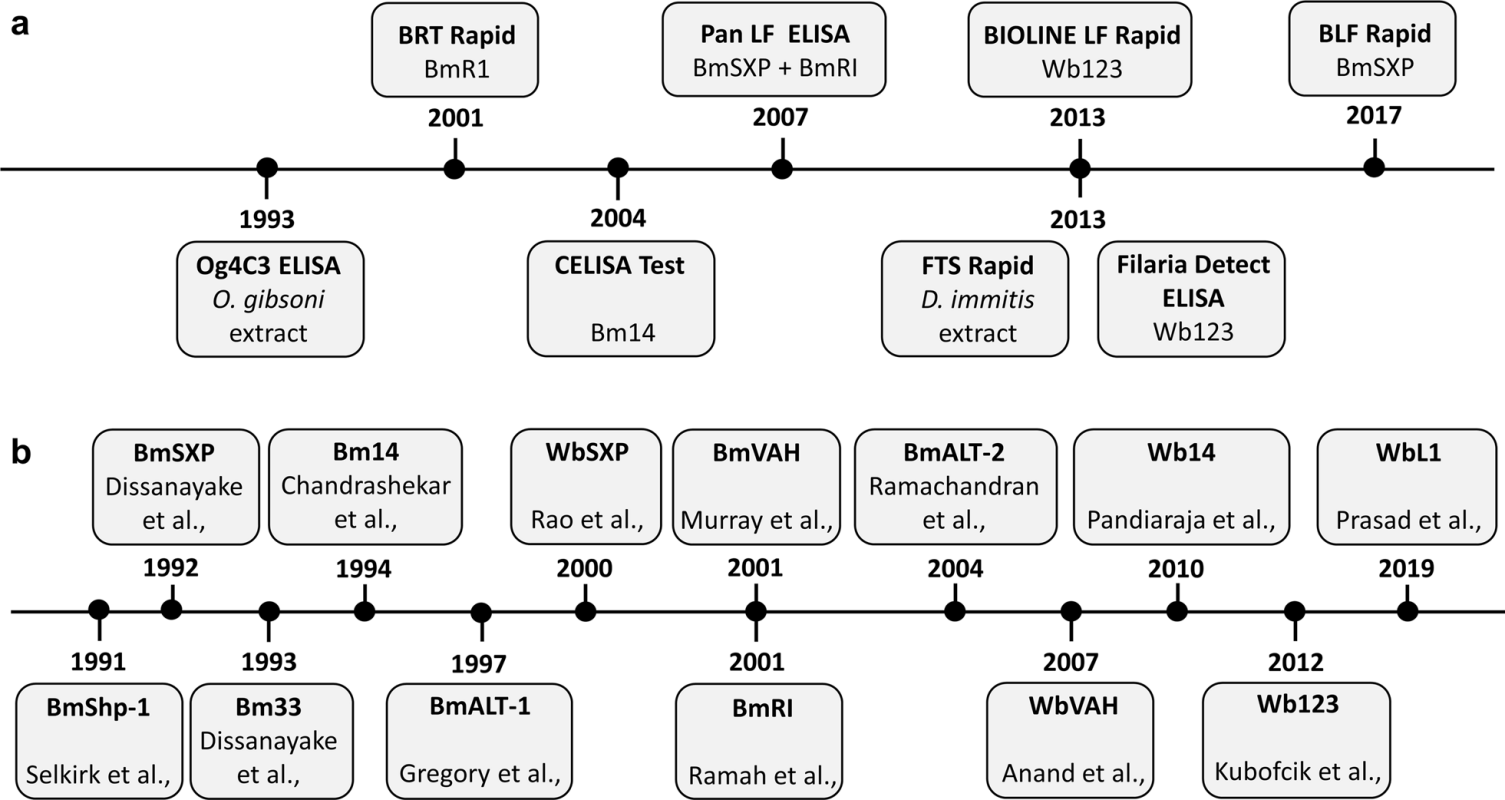
The chronology of lymphatic filariasis commercial tests and recombinant antigens. **a** Main lymphatic filariasis commercially available tests. **b** Recombinant antigens used to develop antibody and antigen capture assays

There are currently eight commercial tests in use for LF diagnosis [15, 17, 21–29]. Two of those, Og4C3 (TropBio^®^, JCU Tropical Biotechnology Pty Ltd, Townsville, Queensland, Australia) and ICT card (BinaxNOW^®^, Abbott Laboratories, Chicago, IL, USA), are based on antibodies produced from worm extracts which are used to capture circulating filarial antigens (CFA). Og4C3 was first developed in 1990 [22], followed several years later by the BinaxNOW filariasis immunochromatographic test (ICT), in 1997 [23]. The latter was replaced by the Alere Filariasis Test Strip (FTS) (Alere, Scarborough, ME, USA) [24, 26]. Six tests are antibody capture assays based on the use of recombinant antigens. These include the CELISA test (Cellabs Pty Ltd., Sydney, Australia) using the Bm14 protein [14], and the Wb123 rapid test (SD Bioline Lymphatic Filariasis IgG4; Standard Diagnostic, Inc., Suwon city, Kyonggi Province, Korea) and Wb123 ELISA (Filaria Detect™ IgG4 ELISA, InBios International, Inc., Seattle, WA, USA), based on the Wb123 antigen [15, 17]. The other antibody capture assays available are the BLF Rapid (Universiti Sains Malaysia—USM), the Brugia Rapid™ test (BRT) (Reszon Diagnostics International Sdn. Bhd., Selangor, Malaysia), and the panLF (Reszon Diagnostics International Sdn. Bhd., Selangor, Malaysia) tests, based on BmSXP, BmR1, or a combination of both recombinant antigens, respectively [21, 28, 29]. In all, several filarial antigens have been produced as recombinant proteins and assayed for possible use in LF diagnosis. Figure 1 summarizes the chronology of the eight main tests available for LF diagnosis, as well as the dates for the first description of the different filarial antigens evaluated for potential use in diagnosis. In the following sections, we will first review the various recombinant antigens described so far, followed by a more detailed analysis of their use for LF diagnosis.

### The SXP/RAL-2 family: BmSXP, Bm14, WbSXP-1, Wb14, and WbL1

The SXP/RAL-2 protein family comprises various related antigens, many independently identified, which have been reported to be useful for LF diagnosis, including two antigens (BmSXP and Bm14) which are the basis of a commercially available diagnostic test. These antigens are encoded by a multi-gene family whose representatives are found in many different nematode species, including *W*. *bancrofti*, *B*. *malayi*, *Onchocerca volvulus*, *Loa loa*, *Ascaris suum*, and *Caenorhabditis elegans*. These proteins are characterized by numerous invariant positions organized into defined motifs [30]. They are best known as potent immunogens and for their importance in diagnosis [19, 30–33]. Although the antigens detailed here appear to be variants of one or a few closely related proteins from *B*. *malayi* or *W*. *bancrofti*, their nomenclature is different, and we will discuss them as they were originally named: BmSXP, Bm14, WbSXP-1, Wb14, and WbL1.

#### BmSXP

BmSXP was first identified through the screening of an expression library made with cDNA derived from adult *B*. *malayi* males and screened with human sera from *W*. *bancrofti*-infected individuals from Sri Lanka. The selected clone encoded a 162 amino acid (aa)-long polypeptide, and the corresponding recombinant antigen was expressed in *Escherichia coli* as a 134 kDa β-galactosidase fusion protein. A rabbit antiserum raised against the recombinant BmSXP identified different bands in Western blots using *B*. *malayi* protein extracts, but most prominently a 14/12 kDa doublet [31].

#### Bm14

Bm14 is a 152 aa recombinant protein whose cDNA was isolated from a *B*. *malayi* cDNA library in an independent serological screening aimed at identifying antigens with potential use for LF immunodiagnosis. The recombinant protein is very similar to BmSXP, with the two cloned fragments differing in four out of 148 aa in their common regions, as well as in their N-terminuses. Antibodies to Bm14 recognized a 13-kDa parasite antigen in *B*. *malayi* protein extracts [19].

#### WbSXP-1 and Wb14

To identify a *W*. *bancrofti*-specific antigen, the BmSXP gene was used to screen a *W*. *bancrofti* L3 cDNA library, leading to the identification of the cDNA encoding WbSXP-1. This cDNA encodes a basic polypeptide with a predicted full-length molecular weight of 20.8 kDa. It differs from BmSXP in having a 29 aa-long C-terminal extension, with the two proteins being 85% identical in the segment which they have in common. Wb14 was derived from the same L3 cDNA library where WbSXP-1 was isolated and is 98% identical to WbSXP-1, even though its C-terminus is similar to BmSXP, missing the 29 aa found in WbSXP-1 [30]. Wb14 is a WbSXP-1 variant, a product of a stop codon introduced at amino acid position 153 and which also differs by three amino acids along their common segment. The WbSXP-1 and Wb14 variants have been shown to be differentially distributed among different *W*. *bancrofti* populations [32]. Searches carried out with available sequences from various worms revealed the presence of homologs to these proteins in many other nematodes with substantial identities in sequence observed in pairwise comparisons. Examples are *O*. *volvulus* (50% identity; Ov-SXP-1), *Ascaris suum* (43%; *As*-SXP-1), *Loa loa* (46%; *Li-*SXP-1), and *C*. *elegans* (29%; *Ce*-SXP-1) [30].

#### WbL1

The last antigen named from this family was WbL1. Its ~ 0.6 kb gene translates into a protein having 153 amino acids and 22.8 kDa molecular weight. It was described as an immunodominant seroreactive clone identified through immunoscreening of a *W*. *bancrofti* L3 cDNA expression library. WbL1 seems to be the same antigen as Wb14 with a single amino acid substitution at position 130, glutamine to leucine [33].

### BmShp-1

The *Brugia malayi* Shp-1 gene was first described as encoding the mf22 protein (BmShp-1). It was isolated through the screening of a mixed adult *Brugia* cDNA library, with polyclonal serum produced against a 29 kDa protein fraction known to be enriched with a previously identified surface glycoprotein. BmShp-1 is a 22 kDa, proline-rich, polypeptide whose expression is upregulated in adult *B*. *malayi* females but not in males. It is also found in the microfilariae, but not in the L3 larvae, where it localizes to the microfilaria sheath, a bag-like structure that envelops the larvae and is a remnant of the embryonic eggshell [34]. BmShp-1 is expressed exclusively in the uterine epithelium of *B*. *malayi* adult females. Based on this localization, it has been suggested that the microfilaria sheath proteins are produced by the uterus and not by embryos [35]. BmShp-1 was found to be the major protein expressed in the microfilaria sheath, considered to be immunogenic, and involved in motility [36].

### Bm33

Bm33 was also discovered through the screening of a cDNA library of male adult *B*. *malayi* worms, but this time with sera from microfilaremic donors infected by *W*. *bancrofti*. This is a pepsin inhibitor with 60% conservation in amino acid sequence with homologous proteins from related organisms, such as the *O*. *volvulus* Ov33 protein. Because of its origin (*B*. *malayi*) and its homology with Ov33, it was named Bm33 [37]. Recombinant Bm33 is an insoluble protein that, when refolded, inhibits the pepsin proteolytic activity. It consists of roughly 85% alpha-helix, and its binding to the human pepsin indicates a 1:1 complex formation [38]. Recombinant rBm33 has been shown to stimulate macrophages to produce a Th1 response but did not induce apoptosis [39]. Immunolocalization of the native protein defined a widespread distribution, both on the surface of the parasite and in internal organs [40].

### BmALT-1 and BmALT-2

These are stage-specific, closely related proteins, found exclusively at the L3 larval stage of the *B*. *malayi* life cycle. Antibodies produced against the recombinant ALT-1 recognized a 22 kDa doublet in soluble L3 extracts. These antigens were originally identified through the finding of their mRNAs as two of the most abundant transcripts from *B*. *malayi* L3 larvae [41, 42]. The BmALT-2 transcript was also found in an immunoscreening of a *B*. *malayi* L3 cDNA library using pooled sera from individuals exposed to *O*. *volvulus* [43] and in a screening of a phage display library with sera from a healthy individual from a *B*. *malayi* endemic area [44]. Recombinant BmALT-1 and BmALT-2 have been evaluated as vaccine candidates [43, 45, 46], and the BmALT-2 immunomodulatory activity has been assessed as a prophylactic tool against diabetes induced by streptozotocin in mice [47].

### BmR1

BmR1 is another *B*. *malayi* antigen whose recombinant version was first reported to be specifically recognized by serum from individuals afflicted with LF [14]. This is a 206 aa-long, 25 kDa polypeptide, with homologs also reported from other parasitic worms. This protein lacks any identifiable domains, and its role is unknown. A secondary structure prediction indicates that it is formed mainly by α-helices, with three epitopes identified as potential antibody binding sites [48, 49].

### BmVAH and WbVAH

The cDNA encoding the *B*. *malayi* version of the venom allergen hormone (BmVAH/VAL-1) was found using the *Ancylostoma caninum* ASP-1 sequence to search for *B*. *malayi* expressed sequence tags (ESTs) derived from the Filarial Genome Project [50]. In addition to BmALT-2, the BmVAH cDNA was also found in a phage display screening with sera from healthy individuals from an endemic *B*. *malayi* area [44]. The BmVAH full-length sequence was found to encode a 232 aa-long polypeptide expressed as a 28 kDa native protein in both microfilaria and L3 stages [50]. WbVAH is its *W*. *bancrofti* homolog, having a sequence identity of 90%, in comparison with BmVAH. Amplification, cloning, and expression of WbVAH using a *W*. *bancrofti* cDNA library from the L3 stage resulted in a 27 kDa recombinant protein [51].

### Wb123

Wb123 is a 372 aa-long protein found in a search for L3 ESTs from *W*. *bancrofti* or *B*. *malayi* having limited similarities to other nematode sequences in public databases. It has been described as a putative serine protease inhibitor that is highly immunogenic in humans [52]. A recombinant protein (GST-tev-Wb123) was expressed in baculovirus and migrated in sodium dodecyl sulphate–polyacrylamide gel electrophoresis (SDS-PAGE), with an estimated molecular weight of 70.4 kDa [17].

## Diagnostic tests

### Proteins of the SXP/RAL-2 family: BmSXP, Bm14, WbSXP-1, Wb14, and WbL1

#### BmSXP

BmSXP was the first recombinant antigen whose reactivity with serum from infected individuals was evaluated (Table 1 summarizes the antigens used to perform the antibody capture assays discussed in this review). In Sri Lanka, a recombinant BmSXP fusion with the maltose-binding protein (MBP) was recognized through enzyme-linked immunosorbent assay (ELISA) by 78% of the microfilaremic sera tested, 16% of sera derived from amicrofilaremic individuals with the acute filarial disease, and 8% of sera from patients with chronic bancroftiasis. This protein was further able to be recognized by microfilaremic sera from individuals from multiple *W*. *bancrofti* endemic areas, such as Tahiti, the Philippines, and Papua New Guinea. It was also recognized by West African onchocerciasis patients and by one individual infected with both *Loa loa* and *Mansonella perstans*. However, oddly, it was not recognized by *B*. *malayi* infected samples. The sera recognition of BmSXP was predominantly through IgG4 subclass antibodies, and it decreased after diethylcarbamazine (DEC) treatment [31]. Subsequently, also using ELISA assays, a polyhistidine (His)-tagged recombinant *Bm*SXP was found to be recognized by 84% and 95%, respectively, of sera from individuals with LF caused by either *B*. *malayi* or *W*. *bancrofti* [21]. Recombinant BmSXP antigen was also used as the basis for a rapid immunochromatographic IgG4 assay, the WB Rapid. This test was evaluated with 489 samples from four countries, with overall sensitivity and specificity greater than 95% [53]. More recently, a related BmSXP-based rapid test, BLF Rapid, was developed and used in Malaysia to assess the prevalence of LF-positive sera in samples from 484 immigrant workers from six countries [28]. It was also evaluated in India, Malaysia, and the USA, showing a sensitivity of 84–100% and 100% specificity [54].

**Table 1:** Antigens used to perform antibody capture assays for the diagnosis of lymphatic filariasis

Antigen	Sensitivity (%)		Specificity (%)	Cross-reactivity	Test used	References
	*Bm*	*Wb*				
BmSXP	0	78	Not shown	Yes (*O*. *volvulus*, *M*. *perstans* and *Loa loa*)	ELISA	[31]
	84	95	99	Yes (*O*. *volvulus*, and *Loa loa*)	ELISA	[21]
	–	97.6	99.6	Yes (other infections)	WB Rapid	[53]
	–	–	–	Not tested	BLF Rapid	[28]
	–	94	100	Not tested	BLF Rapid	[54]
Bm14	90		Not shown	Yes (*O*. *volvulus*)	ELISA	[19]
	–	> 90	Not shown	No (non-filarial helminthiasis)	ELISA	[59]
	91	96	Not shown	Yes (*O*. *volvulus* and *Loa loa*)	CELISA	[15]
	91	98	Not shown	Yes (*Ascaris* and *Strongyloides*)	CELISA	[18]
WbSXP-1	–	100	Not shown	Yes (*Loa loa*)	ELISA	[30]
	90.8	91.4	100	Yes (*Loa loa*)	Rapid test	[65]
	39	91	Not shown	Yes (*O*. *volvulus*, and *Loa loa*)	ELISA	[15]
Wb14	Not shown		Not shown	Not tested	ELISA	[32]
	–	90	96.6	Yes (*Strongyloides*)	ELISA	[62]
(WbT)	–	90	96.6	Yes (*Strongyloides*)	ELISA	[62]
WbL1	–	93	98	Yes (not specified)	ELISA	[33]
Bm33	Not shown		Not shown	Yes (*O*. *volvulus*)	ELISA	[37]
	Not shown		Not shown	Not tested	ELISA	[40]
BmALT-1	Not shown		Not shown	Not tested	ELISA	[41]
BmALT-2	Not shown		Not shown	Not tested	ELISA	[43]
BmR1	96	–	95	No (other infections)	ELISA	[14]
	97	–	99	Yes (other infections)	BmR1 Dipstick	[29]
	100	45	Not shown	No (*O*. *volvulus*, and *Loa loa*)	ELISA	[15]
	100	56.7	Not shown	Yes (*O*. *volvulus*)	BmR1 Dipstick	[15]
	98	14	100	No (*O*. *volvulus*, and *Loa loa*)	ELISA	[21]
BmSXP + BmR1	98	84	99	No (other infections)	ELISA	[21]
	97.2	96	99.6	Yes (other infections)	PanLF Rapid	[53]
BmVAH	Not shown		Not shown	Not tested	ELISA	[50]
WbVAH	Not shown		Not shown	Not tested	ELISA	[51]
Wb123	Not shown	100	100	Yes (*O*. *volvulus*, *Loa loa*)	LIPS	[52]
	–	93	97	Yes (*O*. *volvulus*)	ELISA	[17]
	–	92	96	Yes (*O*. *volvulus*)	Rapid test	[17]
	–	92.6	95.7	Yes (*O*. *volvulus*)	Rapid test	[20]
	Not shown		Not shown	Not tested	Luminex	[80]
Bm Shp-1	Not shown		Not shown	Not tested	ELISA	[83]

Regarding antigen capture tests, a first sandwich ELISA test based on BmSXP was developed using antisera from mice and rabbits immunized with the recombinant antigen. For microfilaremic patients, the test was able to detect 88% (30/34) of sera infected with *W*. *bancrofti* and 83% (25/30) of sera parasitized with *B*. *malayi* (Table 2 summarizes the antigens used to produce antibodies to perform the antigen capture assays discussed in this review). It was also able to show major differences in reactivity with sera from patients with the chronic disease from endemic areas, with 22% (7/31) positivity for individuals from *W*. *bancrofti* areas and no positive results (0/13) seen for those from endemic *B*. *malayi* areas [55]. In order to further optimize the search for specific monoclonal antibodies against BmSXP, phage display technology was used in two independent reports. In the first, an immune scFv library was generated with RNA from the blood of LF-infected donors, resulting in six monoclonal antibodies identified against BmSXP [56]. One of these clones (5B) was used in combination with polyclonal anti-BmSXP on another ELISA sandwich test, resulting in a positive result for all sera assayed from microfilaremic patients infected with *W*. *bancrofti* LF (34/34). This test also showed 100% specificity for diagnosis when tested with sera from 50 healthy individuals and 40 patients with other parasitic diseases, including LF caused by *B*. *malayi* [57]. The second study generated Fab antibodies against *Bm*SXP, selected from a Fab antibody library made with RNA from a pool of B cells that were derived from a large number of healthy blood donors from China, India, and Malaysia. Several clones were selected, with some of those leading to the expression of monoclonal antibodies whose binding to *Bm*SXP was confirmed through ELISA and pull-down assays [58].

**Table 2:** Antigens used to produce antibodies to antigen capture assays for the diagnosis of lymphatic filariasis

Antigen	Sensitivity (%)		Specificity (%)	Cross-reactivity	Test used	References
	*Bm*	*Wb*				
BmSXP	83.3	88	Not shown	No (other parasites)	ELISA	[55]
	–	100	100	No (other infections)	ELISA	[57]
WbSXP-1	80	95	Not shown	No (other parasites)	ELSA	[55]
	–	100	Not shown	Not tested	ELISA	[67]
	Not shown		Not shown	Not tested	ELISA	[68]
	100		Not shown	No (malaria and dengue)	ELISA	[70]
BmVAH	Not shown		Not shown	Not tested	ELSA	[78]
	Not shown		Not shown	No (malaria and dengue)	ELISA	[70]
WbSXP-1 + BmVAH	Not shown		Not shown	Not tested	ELISA	[78]
BmShp-1	Not shown		Not shown	Not tested	ELISA	[84]
BmALT-2	Not shown		Not shown	No (malaria and dengue)	ELISA	[70]

#### Bm14

When first described, the recombinant Bm14 was expressed as a glutathione *S*-transferase (GST) fusion and seen to be recognized by ~ 90% of sera from microfilaremic individuals infected with both *B*. *malayi* and *W*. *bancrofti*, tested using ELISA assays. Cross-reactivity was seen with three of the eight samples from patients with onchocerciasis [19]. A follow-up work confirmed a prevalence of roughly 90% of antibodies against this protein in sera from microfilaremic individuals or those with positive results after testing for filarial antigens. These results contrast with a lack of antibodies against this protein in sera from individuals from non-endemic areas, including those afflicted with non-filarial helminthiasis. Nevertheless, this test did not seem to be able to discriminate between sera from individuals with active infection and from uninfected individuals exposed to the parasite [59]. Subsequently, an IgG4-specific antibody capture ELISA based on Bm14 was seen to produce positive results with samples from patients with different filarial infections, therefore suggesting this as a panfilarial assay. The assay was reactive with sera from patients with *W*. *bancrofti* (91%), *B*. *malayi* (96%), *L*. *loa* (69%), and *O*. *volvulus* (68%) [15]. The GST-Bm14 antigen was also used to monitor antibody prevalence after treatment of bancroftian filariasis with DEC, showing a slow antibody clearance, with ~ 50% positivity remaining 48 months after treatment [60]. In related work, it has been suggested that Bm14 may be useful for monitoring transmission after drug treatment [61]. CELISA is a commercial test that replaced the Bm14 ELISA and has been used as an epidemiological tool to assess levels of infection and exposure to both *W*. *bancrofti* and *B*. *malayi* parasites in endemic regions, although it was also seen to produce positive results in sera from individuals infected with *Ascaris* and *Strongyloides* [18, 62]. CELISA was tested using both dried blood spots and plasma for sample collection, showing no significant differences in positive results using either type of sampling [63, 64].

#### WbSXP-1 and Wb14

Recombinant His-tagged WbSXP-1 was expressed and used to develop an anti-WbSXP-1 IgG4 ELISA assay. This assay was 100% sensitive to sera from patients infected with *W*. *bancrofti*, and produced no positive results with sera from individuals with confirmed *O*. *volvulus*, although a 40% positivity was seen for sera tested from *Loa loa* patients. For this assay, a comparison was carried out with the BmSXP-1 antigen, produced under identical conditions, which showed 88% sensitivity for the *W*. *bancrofti*, but also had positive reactions with sera from both *O*. *volvulus* and *Loa loa* infections [30]. The WbSXP-1 was then used to develop a rapid flow test based on immune filtration and the use of colloidal gold protein A to detect IgG against the recombinant protein. Sensitivity of 91.4% for bancroftian and 90.8% for brugian filariasis was observed in a large trial with 1230 serum samples. Minor reactions were observed with sera from individuals infected with *Loa loa*, but no reactions were seen with *Onchocerca*-positive sera or with sera from individuals with other parasitic diseases, including various diseases caused by protozoans, helminths, and *Schistosoma* [65]. A subsequent study, however, using a rapid cassette test produced based on WbSXP-1, was associated with a much more significant cross-reactivity with both *Loa loa* (43%) and *O*. *volvulus* (60%) sera [15].

An ELISA sandwich assay using polyclonal antibodies produced against WbSXP-1 in mice and rabbits was developed as an antigen capture test. For bancroftian filariasis, 95% of microfilaremic sera plus 10% of sera associated with chronic pathology and 3% of sera from uninfected individuals from endemic areas were positive. For brugian infection, however, the assay was positive only for 80% of the microfilaremic sera, with no positive results with equivalent sera from the other two groups [55]. A subsequent study used monoclonal antibodies against WbSXP-1 to develop a more robust and specific assay. The antibodies recognized the recombinant antigen as well as the native protein from microfilaria extracts of both *W*. *bancrofti* and *B*. *malayi* and could also react with sera from individuals infected with the two parasites. For a preliminary ELISA sandwich assay, a polyclonal rabbit anti-WbSXP-1 serum was used for the capture antibody and one of the monoclonal antibodies (1AC62) was used for the detection, with the assay being able to detect circulating antigen from both worms [66]. A second assay used a new set of monoclonal antibodies for the capture step and a polyclonal rabbit anti-WbSXP-1 serum for detection. Here, bancroftian filariasis samples were analyzed, with 100% of the microfilaremic sera and 14% of the sera from healthy individuals from endemic areas testing positive, while sera from patients with chronic pathology or healthy controls from non-endemic areas did not show reactivity [67]. This assay was used as the basis for an evaluation of a new method of sample collection, where 100–150 µl aliquots of blood were collected directly through a smear on a microscopic slide. This smear was allowed to dry for storage and was subsequently resuspended in phosphate-buffered saline (PBS), before using the resuspended sample for the filarial antigen detection. When compared with standard sera or whole blood collection, the new method did not show significant differences in optical density (OD) values for the assay results. Furthermore, the rWbSXP-1 antigen assay was responsible for a greater than fivefold increase in positivity amongst a large field survey in an endemic area, when compared with the conventional microscopic staining method, presumably allowing the identification of a large number of false-negative cases [68].

Specific WbSXP-1 peptides were evaluated as alternatives to the full-length recombinant antigen for diagnostic purposes. To this end, four peptides derived from the WbSXP-1 sequence, and predicted to encompass immunodominant B-cell epitopes, were chemically synthesized and tested individually or in combination against human clinical sera from LF individuals. Chimeric peptides consisting of two of the four peptides, in different combinations and linked in tandem, were also synthesized and evaluated. The best results were seen for the first three peptides [69], found in the sequence that WbSXP-11 has in common with Wb14, the truncated version of WbSXP-1 [32].

A study investigated the use of a recombinant His-tagged Wb14 for its potential to be recognized by total human IgG from different LF sera. In preliminary assays, Wb14 performed similarly to WbSXP-1 with different sets of sera, including those from microfilaremic individuals, although the data shown indicate that WbSXP-1 is more reactive than Wb14 [32]. Recently, a variant protein named WbT was generated through the removal of the hydrophobic, 17 aa-long N-terminus of Wb14. This was intended to facilitate recombinant protein expression and humoral recognition, but no differences in bacterial expression or antibody recognition through ELISA assays were seen between WbT and Wb14. Indeed, both anti-Wb14 and anti-WbT IgG4 capture assays were performed with similar sensitivity (90%) and specificity (96.6%) as the standard Og4C3 and POC-ICT tests, when evaluated with sera from patients with bancroftian LF. Nevertheless, WbT and Wb14 did perform with higher specificity when compared with the CELISA test (70%) based on the recombinant Bm14 [62].

#### WbL1

The *W*. *bancrofti* antigen WbL1 was recently chosen as the antigen for an ELISA aiming to diagnose LF based on IgG and IgG4 detection. The anti-IgG ELISA recognized ~ 69% of the microfilaremic sera tested and 35% of sera from patients with clinical bancroftian filariasis. The anti-IgG4 assay exhibited better performance for the microfilaremic sera, with 77% and ~ 86% positive results, respectively, for a first optimized analysis and a subsequent multicentric validation study, with up to 50% positivity for the patients with clinical filariasis in the multicentric study. This multicentric evaluation displayed a maximum sensitivity of 93% and specificity of 98%. The ELISA anti-WbL1 IgG4 was then proposed as a new optional test for initial screening and epidemiological surveys of filarial infections in LF endemic areas [33].

### Bm33

In an early study, the immune response by different sera against a recombinant Bm33, expressed as a β-galactosidase fusion, was evaluated using ELISA assays. This study revealed that Bm33 was recognized by 71% of microfilaremic sera from Sri Lanka individuals infected with *W*. *bancrofti*. Conversely, only 12% of sera from individuals infected with *O*. *volvulus* and none of the sera infected with either *Mansonella* or *Loa loa* were positives. These results were similar to those seen with the BmSXP-1 MBP fusion but differed from the results seen for Ov33, the recombinant Bm33 ortholog from *O*. *volvulus*. Ov33 was recognized by sera from only 24% of microfilaremic individuals infected with *W*. *bancrofti*, despite producing a positive result with 90% of the sera from patients infected with *O*. *volvulus*. These species-specific differences seen between Bm33 and Ov33, however, cannot be easily explained by the limited differences seen when the sequences of the two antigens are compared [37]. Subsequently, Bm33 was expressed as an MBP fusion and used in an investigation with sera from both microfilaremic patients and amicrofilaremic individuals from an endemic area in Indonesia. This study demonstrated a high IgG4 and IgG1 response against the recombinant Bm33 [41]. More recently, individuals from Chennai, an endemic region in India, were seen to produce an IgG response against a His-tagged recombinant Bm33, with the highest positivity seen for microfilaremic sera, followed by sera from chronic patients and healthy controls from the endemic area, with no reaction with sera from healthy individuals from non-endemic areas. For this study, an isotype-specific analysis showed elevated levels of IgG4 and IgE, especially for the microfilaremic sera, although no statistically significant difference could be defined for the three groups from the endemic area, microfilaremic, with chronic pathology, or asymptomatic and amicrofilaremic [40].

### BmALT-1 and BmALT-2

A recombinant version of the BmALT-1 antigen, expressed with a C-terminal His-tag, was also used to analyze antibody response in LF individuals. Humans exposed to *B*. *malayi* from endemic areas, amicrofilaremic or microfilaremic had significantly higher levels of circulating IgG1 and IgG3 antibodies against BmALT-1 and little or no response associated with IgG4. These results contrast with what was seen with Bm33 and other recombinant antigens, where IgG4 antibodies are generally seen to be associated with the strongest response [41]. In an independent study, recombinant BmALT-2 was expressed with an N-terminal His-tag and also evaluated with sera from individuals from a *B*. *malayi* endemic area. Remarkably, much higher positivity (72%) was seen for the sera from healthy individuals than for the microfilaremic sera (36%) or the sera from patients with chronic lymphatic pathology (52%). The authors proposed that a protective immunity for the uninfected individuals might have been associated with a stronger response to BmALT-2 [43]. Indeed, the strong reactivity of BmALT-2 mostly with sera from healthy individuals from an endemic area was further confirmed in a second study [44].

More recently, antigen capture ELISA sandwich assays were optimized using specific monoclonal antibodies and polyclonal sera raised against the recombinant BmALT-2. The best combination used a polyclonal serum plus one of the monoclonal antibodies to capture the antigen and another monoclonal antibody for detection. This test was not able to produce positive results with sera from microfilaremic patients, contrasting with the WbSXP-1 (described above) and VAH (see below) capture assays that were positive for all microfilaremic individuals. Nevertheless, the ALT-2 assay produced positive results with more than half (57%) of the sera from healthy individuals living in an area of high filarial incidence, comparable to the VAH test (52%), with WbSXP-1 producing no positive results with these sera [70].

### BmR1

The recombinant BmR1 was first evaluated for diagnostic purposes in an immunoassay to detect IgG4 antibodies in sera from patients infected with *B*. *malayi*, with the results showing a sensitivity of 96%, with 95% specificity [14]. Results published almost simultaneously also described the BmR1 antigen as the basis for the BRT dipstick test, which showed a sensitivity of 97%, with 99% specificity [29]. Subsequently, the BmR1 rapid assay was used in a multicentric evaluation with a very large number of sera to better evaluate its use for the diagnosis of brugian filariasis. Sensitivity of over 90% for microfilaremic sera was observed in tests carried out by three different laboratories, based in India, Switzerland, and the Netherlands [71]. The BmR1 rapid test was also used to detect antibodies in filariasis patients infected with *B*. *timori*. It was seen that 100% of patients who had microfilaria reacted with the BmR1 and 76% of patients who did not have microfilaria (symptomatic and asymptomatic) were also reagents [72].

Three different assay formats based on BmR1 (ELISA, dipstick, and cassette) were used in a study aiming to compare their efficiency for LF diagnosis with other recombinant antigens (Bm14 and WbSXP-1). The BmR1-based tests were the most efficient for *B*. *malayi*, with 100% sensitivity, despite a much poorer performance with the sera derived from *W*. *bancrofti* patients (45%). Furthermore, the BmR1 assay was remarkably specific for the *W*. *bancrofti* and *B*. *malayi* infections, showing either little reactivity (0–5%) with samples from people with *O*. *volvulus* or no reactivity with sera from individuals infected with *Loa loa* or other helminths (*Strongyloides*) [15]. Another study compared the BmR1 rapid test with the ELISA using soluble worm antigen (SWA-ELISA) in order to demonstrate the prevalence of IgG4 antibodies against *B*. *malayi*, with similar results [73]. The antibody response to recombinant BmR1 was also compared with the response to its homologs from related helminths (*W*. *bancrofti*, *O*. *volvulus*, and *L*. *loa*), with a similar response seen from individuals infected with the different parasites against the corresponding recombinant proteins [49]. The BRT test was also evaluated as a tool to monitor the prevalence of anti-filarial IgG4 antibodies after mass drug administration in filariasis endemic areas, confirming that it was possible to detect persistence of anti-filarial antibodies after the disappearance of microfilaria [74]. More recently, this test was used to monitor the incidence of lymphatic filariasis in three districts of Indonesia [75]. WHO currently indicates the commercially produced BRT antibody-detection test (Reszon Diagnostics International, Subang Java, Selangor, Malaysia) for the monitoring and evaluation of LF in *Brugia* spp. areas [24].

In a study aimed at developing a single assay capable of detecting antibodies against the different types of LF, recombinant BmR1 and BmSXP were compared on their own or combined in a mixture of both antigens (1:1). For the detection of brugian filariasis, sensitivity of 98% was seen for BmR1 alone or combined with BmSXP, compared with the 84% sensitivity seen for BmSXP alone. In contrast, for bancroftian filariasis, the assay based on BmSXP alone was more sensitive (95%) than an assay using only BmR1 (14%) or a mixture of these two antigens (84%) [21]. These results motivated the development of a test using both antigens (panLF Rapid) on the same platform for filariasis diagnosis, with performance of 96% sensitivity and 99% specificity [21, 53]. The panLF test has been used to evaluate the efficacy of large-scale LF treatments based on mass drug administration [76, 77].

### BmVAH and WbVAH

A His-tagged recombinant BmVAH/VAL-1 was also used in ELISA assays to assess the immune response to this protein with sera from individuals with confirmed microfilaria from a *B*. *malayi* endemic area and from healthy controls. High levels of IgG3 and IgG4 were generally observed, with 95% (20/21) and 86% (18/21) positivity seen for the microfilaremic and healthy groups, respectively [50]. Subsequently, healthy individuals from an endemic area were independently confirmed to be carriers of circulating antibodies against BmVAH/VAL-1 [44]. More recently, polyclonal sera and monoclonal antibodies were also produced against the BmVAH and used to develop another ELISA sandwich assay, called VAH ELISA, for the detection of filarial antigen. The test, based on a combination of polyclonal sera for the capture step and a biotinylated monoclonal antibody for the detection, identified ~ 98% or 100% of microfilaremic individuals infected either with *W*. *bancrofti* or *B*. *malayi*, respectively. When the VAH ELISA was combined with the WbSXP-1 capture ELISA (described previously), 100% of the microfilaremic individuals infected with either of the two parasites were detected, with enhanced reactivity [78].

As for the WbVAH antigen, a recombinant His-tagged protein was used to study the presence of antibodies in microfilaremic individuals and those with chronic pathology in ELISA assays. The best results were seen for healthy individuals from an endemic area, with a response based mainly on IgG1, IgG2, and IgG3 circulating antibodies [51].

### Wb123

The identification of Wb123 as a relevant recombinant antigen for LF diagnosis was based on the results produced using it as the basis for a Luciferase Immunoprecipitation System Platform (LIPS) assay [79]. In this assay, Wb123 was expressed in mammalian cell infusion with Renilla luciferase (Ruc) and incubated with the sera to be tested and protein A/G beads. The binding of Ruc-Wb123 to the beads is dependent on the presence of antibodies against Wb123 in the sera, with the fusion protein detected by assaying the luciferase activity. The assay was shown to have a sensitivity of 100% with sera from *W*. *bancrofti* patients, with minor cross-reactivity seen with sera from infections with *B*. *malayi*, *L*. *loa*, and *O*. *volvulus* [52]. The LIPS Wb123 assay was also used to evaluate children from Mauke, Cook Islands, born 5 years after mass drug treatment. A positive correlation was found between a reduction in the prevalence of anti-Wb123 antibodies and reduced transmission [17].

With its efficiency on the LIPS platform confirmed, a GST-Wb123 fusion was expressed in the baculovirus system in insect cells and used as the basis for both an ELISA and a lateral-flow immunoassay, both tested for LF diagnosis. The two tests were performed very efficiently with sera from patients infected with *W*. *bancrofti*, with similar sensitivity (93% for the ELISA and 92% for the rapid immunoassay) and specificity (97% and 96%) and minor cross-reactivity seen only with sera of individuals infected with *O*. *volvulus* [17]. With the availability of the *O*. *volvulus*-specific Ov16 antigen, and considering the overlap in the incidence of both *W*. *bancrofti* and *O*. *volvulus* in African countries, both Wb123 and Ov16 antigens were evaluated as part of a single rapid test designed for the simultaneous diagnosis of both bancroftian filariasis and onchocerciasis. Sensitivity higher than 90% was observed for the two antigens, with the results equivalent to those seen with tests based on a single antigen, confirming the utility of the test for the diagnosis of both diseases in endemic regions [20]. Both Wb123 and Ov16 were also used in multiplex bead assays (Luminex) to assess the reactivity of antibodies against these antigens, and determine disease prevalence, in sera from individuals from three Senegalese endemic regions [80]. The efficiency of the use of Wb123 in diagnostic tests was further confirmed in a comparison between the more traditional ICT and Og4C3 ELISA tests with the Wb123 ELISA, in a surveillance study aiming to evaluate the prevalence of filarial antibodies after mass treatment, with similar results observed for the three tests [81]. Furthermore, when evaluated with a large set of sera from individuals with confirmed infection with *Loa loa* in Cameroon (Africa), several with high microfilaria load, rapid tests based on Wb123 were found to produce little or no false-positive results [82]. Currently, several commercial tests for LF diagnosis use the recombinant Wb123 antigen, including the Wb123 rapid test only (Bioline Lymphatic Filariasis IgG4); Wb123 ELISA (Filaria Detect™ IgG4 ELISA), and Ov16 + Wb123 rapid test (Bioline Oncho/LF IgG4).

### Bm Shp-1

Although first studied in the early 1990s, the *B*. *malayi* sheath protein (BmShp-1) has only more recently been evaluated for its use as a diagnostic tool, with the realization that its repeat region, encompassing amino acid residues 49 to 107, includes dominant B epitopes. Both this protein fragment and the full-length polypeptide, bacterially expressed with a His-tag, were used in ELISA assays to investigate the presence of anti-BmShp-1 antibodies in sera from individuals from a filarial endemic population. Positive results were seen for both proteins, with no significant differences in performance between them, confirming the role of the repeat region in inducing an immune response. Interestingly, the sera from individuals from the endemic region who lacked microfilaria in the blood, and were asymptomatic, were associated with a higher reactivity than the sera from microfilaremic individuals or those with chronic pathology, with no reactivity seen for healthy controls from non-endemic regions [83].

Polyclonal sera and monoclonal antibodies were also produced against the full-length BmShp-1 and used in an ELISA sandwich assay evaluated as an alternative antigen capture test. Using the polyclonal serum for the capture step and the biotinylated monoclonal as a detection antibody, all patients from microfilaremic groups infected with either *W*. *bancrofti* or *B*. *malayi* were positive with the assay. Furthermore, when compared with two other ELISA sandwich assays, WbSXP-1 and Og4C3, only the anti-BmShp-1 ELISA gave positive results for healthy individuals from the endemic area (12%) and those with chronic pathology (29%), highlighting the potential use for this assay in monitoring the effectiveness of mass drug administration [84].

## Assays recommended in the GPELF: advantages and disadvantages

WHO recommends three assays for LF diagnosis in the GPELF [24]. Circulating microfilariae are best identified by examining thick smears (20–60 μl) of finger-prick blood, the Alere FTS (Filariasis Test Strip) is recommended for detecting *W*. *bancrofti* antigens on human blood samples, and the BRT assay (Brugia Rapid point-of-care cassette test) is the proposed alternative for the detection of IgG4 antibodies against *Brugia* spp. in human blood samples.

Each assay has advantages, but also minor issues that might prevent adequate use or impact its effectiveness. The parasitological test is cheap and highly specific; however, its sensitivity is low, possibly leading to false-negative results, and may require late night collection times, an inconvenience that might prevent adequate sampling [11, 12]. The easy-to-perform FTS is a qualitative low-cost test that avoids the need for the laboratory infrastructure required in parasitological assays or ELISA [26, 85], and it has thus been employed to perform LF transmission assessments in field surveys [26, 86]. Although FTS is used in the GPELF to detect circulating filarial antigen (CFA), it may appear 1 year or more after infection and persists quite a few years after adult worms have died or no longer reproduce [87]. As for the BRT assay, rapid and efficient, it is associated with cross-reactivity with other parasites causing non-lymphatic filariasis (non-LF), such as *O*. *volvulus* [15], although this is not necessarily a problem because non-LF generally does not occur in *Brugia* endemic areas [88].

To direct the surveillance activities for monitoring LF presence or incidence after post-elimination attempts based on mass drug administration, the GPELF needs diagnostic tests for the detection of low levels of *W. bancrofti*, *B. malayi*, and *B. timori*. WHO has therefore created a document called a target product profile (TPP) that describes the minimum and ideal features desired for new diagnostic tools. In summary, the ideal test must target filarial molecules related to recent exposure to be applied in areas under surveillance to better outline the status of infection and/or transmission [87]. These issues have to be kept in mind in efforts to improve upon the current diagnostic tests available.

## Conclusions

As discussed in the current review, the use of recombinant antigens has greatly increased the number of options available for LF diagnosis based on antigen and antibody capture assays. Taking into account the commercially available antibody capture tests, and in addition to the BRT assay based on the BmR1 antigen [75], five others tests are considered as options for LF diagnosis. These are the BLF Rapid, produced using BmSXP [29]; the CELISA, based on the recombinant Bm14 [18]; the IgG4 ELISA and rapid test, manufactured using the Wb123 antigen [17]; and the panLF, capable of detecting antibodies in patients infected by *Brugia* or *Wuchereria* species [21]. Regarding antigen capture tests, however, the two most widely used tests are still derived from antibodies raised against protein extracts from worms that do not cause human LF, the FTS rapid test, and the Og4C3 ELISA [22, 23]. The increase in the use of tests based on recombinant antigens for LF diagnosis has the potential to solve the stated limitations related to cross-reactivity and low sensitivity. Further technological advances, as reported in some of the most recent studies using phage display and the BmSXP and BmRI antigens [27, 56, 58], have the potential to facilitate the generation of more efficient antibodies that can be used for such new assays. Another possibility is the use of tests based on chimeric proteins which can potentially enhance the capabilities of the antibody capture assays, as has been done for other diseases [89–91]. Thus, along with what has been reported so far, it is expected that further progress (or advances in research) should facilitate the development of a test that combines the features of high sensitivity, high specificity (without cross-reactivity), and low cost, to assist GPELF.

## Supplementary Information

**Additional file 1: Text S1.** Search strategy and article selection criteria.

## Data Availability

All articles from which data were used to support the conclusions of this review are cited in the text and the reference list.

## Abbreviations

aa: Amino acids
BRT: Brugia rapid test
CFA: Circulating filarial antigens
DEC: Diethylcarbamazine
ELISA: Enzyme-linked immunosorbent assay
EST: Expressed sequence tags
FTS: Filariasis Test Strip
GPELF: Global Programme to Eliminate Lymphatic Filariasis
GST: Glutathione *S*-transferase
ICT: Immunochromatographic test
LF: Lymphatic filariasis
LIPS: Immunoprecipitation system
MBP: Maltose-binding protein
OD: Optical density
PBS: Phosphate-buffered saline
WHO: World Health Organization
