# Supplementary material for: Recombinant antigens used as diagnostic tools for lymphatic filariasis
Source: Parasit Vectors. 2021 Sep 15;14:474. doi: 10.1186/s13071-021-04980-3 (PMC8442287; doi:10.1186/s13071-021-04980-3)
Supplement: Supplementary file 1 — Additional file 1: Text S1. Search strategy and article selection criteria. [file 13071_2021_4980_MOESM1_ESM.docx]

**Additional file 1: Text S1. Search strategy and article selection criteria.**

This study is a semi-systematic review [1] carried out using papers that described or dealt with the use of recombinant antigens for the LF diagnosis. We made a thematic analysis to provide a historical overview of those recombinant antigens, highlighting their advantages and limitations as well as the commercial tests developed based on them.

All papers used to support this review are available on PubMed ([https://pubmed.ncbi.nlm.nih.gov)](https://pubmed.ncbi.nlm.nih.gov/). The papers included complied with at least one of the following criteria: i) Statement related to how an antigen was discovered; ii) Description of the protein structure and/or functions; iii) Report on the first use of an antigen in immunological tests to detect LF, made in house or commercially; iv) Description of combinations of antigens for the LF diagnosis; v) Relate to results with high impact information regarding the LF diagnosis area.

We left out papers based on the following exclusion criteria: i) Review on LF molecular diagnosis; ii) Description of consecutive uses of the same antigens cited before; iii) Description of the use of a recombinant antigen only for purposes other than diagnostic.

**Reference**

1. Snyder H. Literature review as a research methodology: An overview and guidelines. J Bus Res. 2019;104:333–9.
